# Visible and Thermal Image-Based Trunk Detection with Deep Learning for Forestry Mobile Robotics

**DOI:** 10.3390/jimaging7090176

**Published:** 2021-09-03

**Authors:** Daniel Queirós da Silva, Filipe Neves dos Santos, Armando Jorge Sousa, Vítor Filipe

**Affiliations:** 1 INESC Technology and Science (INESC TEC), 4200-465 Porto, Portugal; filipe.n.santos@inesctec.pt (F.N.d.S.); asousa@fe.up.pt (A.J.S.); vfilipe@utad.pt (V.F.); 2 School of Science and Technology, University of Trás-os-Montes e Alto Douro (UTAD), 5000-801 Vila Real, Portugal; 3 Faculty of Engineering, University of Porto (FEUP), 4200-465 Porto, Portugal

**Keywords:** deep learning, forest mobile robotics, forest trunk detection, object detection, SSD, SSDLite, YOLO

## Abstract

Mobile robotics in forests is currently a hugely important topic due to the recurring appearance of forest wildfires. Thus, in-site management of forest inventory and biomass is required. To tackle this issue, this work presents a study on detection at the ground level of forest tree trunks in visible and thermal images using deep learning-based object detection methods. For this purpose, a forestry dataset composed of 2895 images was built and made publicly available. Using this dataset, five models were trained and benchmarked to detect the tree trunks. The selected models were SSD MobileNetV2, SSD Inception-v2, SSD ResNet50, SSDLite MobileDet and YOLOv4 Tiny. Promising results were obtained; for instance, YOLOv4 Tiny was the best model that achieved the highest AP (90%) and F1 score (89%). The inference time was also evaluated, for these models, on CPU and GPU. The results showed that YOLOv4 Tiny was the fastest detector running on GPU (8 ms). This work will enhance the development of vision perception systems for smarter forestry robots.

## 1. Introduction

In recent years, the development of robotic solutions to operate in forestry areas is becoming increasingly more important due to the regular appearance of wildfires. This calamity is mostly triggered by poor management of forest inventory. With this in mind, we study and compare several Deep Learning (DL) models for the purpose of forest tree trunk detection. Then, the models can be utilized for autonomous tasks or inventory-related tasks in forest contexts.

Forestry mobile robotics is a developing domain which has been growing in the last decade. In 2008, BigDog, one of the first autonomous quadruped robot capable of walking in rough and challenging terrains, appeared [1]. This robot was made of about 50 sensors just to control its body motion. The authors reported that its longest continuous operation lasted 2.5 h and consisted of a 10 km hike. Initially, this robot was controlled by a human operator through a remote controller, but in 2010, the authors revealed an update most related to the autonomy of BigDog [2]. To achieve the highest levels of autonomy, the authors nurtured BigDog with a laser scanner, a stereo vision system, and navigation algorithms, that enabled the robot to “see” its surroundings, detecting forest bio-products such as trees and boulders, and steering itself to avoid these obstacles. This resulted in BigDog performing autonomous navigation between goal positions in rough forest terrains and unstructured environments with a high rate of success: the robot reached the goal positions in 23 of 26 experiments, and one time it travelled about 130 m without operator interference [2]. Another self-navigated Unmanned Ground Vehicle (UGV) appeared in 2008 [3], in which the authors developed a control system on top of the Learning Applied to Ground Robotics system developed at Carnegie Mellon. The UGV performed three runs in extreme conditions and on harsh forest terrain, and completed the three successfully in 150 s, with courses of on average 65 m and the UGV maintaining an average speed of at least 0.43 m/s. Thus, the system outperformed the baseline method, proving its robustness [3]. In 2010, a study was conducted on visual guidance for autonomous navigation on rain forest terrain [4], where the authors concluded that visual perception is a key factor for autonomous guidance in forests, despite some limitations that still exist, such as uneven terrain and illumination, false positives, water bodies and muddy paths, and unclassified terrain. Additionally, the authors also tested some approaches to tackle the previous issues such as the detection of water puddles, ground, trees and vegetation [4]. Autonomous navigation in forests not only aims at small-scale vehicles, but also at high-payload machines such as forwarders, as the authors in [5] indicate. The autonomous forwarder weighs around 10,000 kg and was equipped with a high-precision Real-Time Kinematic (RTK) Differential Global Positioning System (GPS) to measure the vehicle position and heading and a gyroscope to compensate for the influence of the vehicle’s roll and pitch [5]. All this setup was then used to make the forwarder follow paths. In such tasks, the vehicle tracked two different paths, three times each, presenting average tracking errors of about 6 and 7 cm; the error never exceeded 35 cm and, in most parts of the paths, the error was less than 14 and 15 cm. Another application in a forwarder was carried out in [6]. In this work, the authors proposed a method that detects trees (by means of classification) and computes the distance to the trees for autonomous navigation in forests. For tree detection, the forwarder was equipped with a camera and machine learning classifiers—artificial neural network and k-nearest neighbours—were used along with colour and texture features. Such a combination of features resulted in high classification accuracies. The proposed method for measuring the distance to the trees works fairly well if the ground is flat and if there are no overlapping objects in the scene [6]. Another vehicle that was tested for the task of autonomous navigation was a robotic mower in 2019 [7]. In that study, the authors developed an autonomous navigation system using visual Simultaneous Localization And Mapping (SLAM) and a Convolutional Neural Network (CNN), without GPS. The system was tested only on forward and backward movements, and the results were comparable with the ground-truth trajectories [7].

Autonomous navigation in forests is not only possible on land with UGVs, but also in the air with Unmanned Aerial Vehicles (UAV). In 2016, a UAV with GPS, an inertial measurement unit and a laser scanner was developed along with Kalman filter and GraphSLAM techniques to achieve autonomous flights in the forest [8]. An interesting approach was taken in [9], where the authors proposed a method for autonomous flight of a UAV based on following footpaths in forests. Using CNNs, the system was able to locate the footpaths and select which one to follow, in case there are several, using a decision making system. An update of an existing algorithm for UGVs, which calculates the distance to obstacles [10] using image features, has been proposed in [11]. In this work, the algorithm was used for UAVs and this new version of the algorithm provided significant improvements relative to the original, working at 15 frames/s for frames with a size of 160 × 120 pixels [11]. Another UAV-based work aiming for autonomous flying was presented in [12]. In this work, an autonomous flight system was proposed that carries out tree recognition using a CNN and then generates three possible results: free space, obstacle close and obstacle very close. When the latter two are generated, the UAV “decides” which side must be picked to perform the evasion manoeuvre. The system was tested in a real environment and both tree recognition and the avoidance manoeuvre were carried out successfully [12].

In agricultural contexts, there are some interesting works that combine mobile robotics with visual systems. In [13], the authors proposed a system for phenotyping crops composed of two parts: a terrestrial robot responsible for data acquisition and a data processing module that performs 3D reconstruction of plants. The robot was mounted with an RGB-D camera that captured images for further 3D reconstruction processing. The authors concluded from the experiments that both a plant’s structure and environment can be reconstructed for health assessment and crop monitoring. Another robotic platform that was developed to monitor plant volume and health was introduced in [14]. Here, the authors proposed a robotic platform equipped with lasers and crop sensors to perform reliable crop monitoring. The experiments showed that the reconstruction of a volume model of plants was successfully performed and the plants’ health was assessed by using the crop sensors through the calculation of the normalized difference vegetation index. Then, the volume-based model was merged with crop sensors’ data, enabling one to determine the vegetative state of plants. A similar work to the previously mentioned one has been proposed in [15]. In this work, the combination of laser data with crop sensors’ data was used to detect plant stress and to map the vegetative state of plants. In [16], the authors proposed a deep learning-based method for counting corn stands in agricultural fields. A handheld platform was used to mount and test the hardware and software pipeline, which the authors claim that can be easily mounted on carts, tractors or field robotic systems. The pipeline utilizes a YOLOv3 architecture to detect the corn plants and a Kalman filter to track and count the plants. The plant counting was performed with high accuracy.

In forestry and agricultural mobile robotics, the robot visual perception of the environment is a matter of the utmost importance, where several challenges can appear, such as trees, bushes, boulders, holes and rough terrain (with mud, rocks, etc.). In the agricultural context, there are plenty of works and studies related to image-based woody trunk detection, specially for performing SLAM [17,18,19,20]. On the other hand, in the forestry context, there are some works that focused on image-based forest tree detection. In [4], the authors present a preliminary study on the detection of forest products such as green vegetation, tree trunks, ground and water bodies. For this, they used a colour camera and stereo camera, and through hue, saturation and value histograms they were able to distinguish the aforementioned forest products. However, the authors did not present any numerical results for further comparison with other works. In [9], an autonomous UAV is proposed that relied on the detection of footpaths by a CNN so that the UAV could follow them. Again, there are no numerical results in terms of trail detection or recognition in the paper. In a similar way, a deep learning approach for flying autonomously in forests was presented in 2018 [12]. The authors used a modified version of the AlexNet [21] CNN to classify frames. Based on the proximity of existing objects, the network generated one of three different outputs (classes): free space, obstacle is close, or obstacle is very close. With these outputs the UAV performed some actions to avoid the objects. The CNN was trained on a dataset formed by 9500 simulated and real images for each class. To test the CNN, 100 flights were made in a simulated environment and 10 in a real environment. The results presented 85% and 100% success rates in the simulated and real environments, respectively. In [6], the authors propose a vision application to drive a forwarder autonomously in the forest. On top of the forwarder, a Charge-Coupled Device (CCD) camera was mounted to acquire the forest images. Each image was analyzed in order to find forest tree trunks by combining colour spaces and image descriptors with two classifiers—K-Nearest Neighbours (KNN) and Artificial Neural Network (ANN). After the trunks were detected, they were segmented, the distance to them was measured, and, if the distance was below a proximity threshold, the vehicles stopped and waited for a command from the human operator; otherwise, the vehicle continued the operation and the acquisition of images. The results showed that KNN achieved better classification accuracy in all colour space-feature combinations than ANN, with the highest accuracy being 94.7%. In [22], the authors performed automatic tree detection using street images using YOLOv2 with two different feature extractors: ResNet-50 and a four-layer CNN. They tested the models in a test dataset composed of 69 trees and they claimed it could achieve 93% and 87% accuracies in the same dataset with ResNet-50 and the four-layer CNN as feature extractors, respectively. The detection of trees in street images was also carried out in [23]. In this work, the authors proposed a part attention network for tree detection based on Faster R-CNN [24]. To assess their approach they used a dataset composed of 2919 manually labelled images, from which 500 were used to test the method, comprising 1464 trees. The best (lowest) miss rate achieved was about 20.62%. The authors compared their method against four well-known deep learning architectures, resulting in their method being superior to such models. Another work related to forest tree trunk detection, which uses fuzzy logic combined with a contour transform was proposed in [25]. In this work, a CCD image is fused with an infrared image to segment tree trunks in forests. The proposed method was evaluated against other methods using nine metrics. The authors concluded that their method is better at describing the reality of a scene and can be used for real-time applications. Despite these works, more research is need, as there are no significant studies about this topic at ground level which focus on the detection of tree trunks with deep learning models and their evaluation with well-known metrics in the object detection domain. Furthermore, the majority of works related to forest tree detection are focused on performing the detection with Light Detection and Ranging (LiDaR) data alone [26,27,28,29,30], with aerial high-resolution multispectral imagery alone [31,32,33,34,35,36,37,38,39,40] or with a combination of both [41,42,43,44,45].

As mentioned above, the detection of forest tree trunks can be made with data from LiDaRs or image-based sensors. The advantages of LiDaR are: it provides 3D information about a scene and, depending on the type of LiDaR, normally its data do not suffer from light variations (in contrast to cameras). The most important advantages of cameras are: they can be much less expensive than LiDaRs and they have much more resolution and information on the field of view. In some cases, depth information can also be acquired by multiple camera systems (for example, stereo vision), making the robot aware of the distance to a point of interest.

In this work, we intend to further develop the domain of detection of forest tree trunks by studying such detection with visible and thermal images to enable performing of autonomous tasks, for navigation and inventory purposes, in forests during the day and night. The night-time operational context of this study is particularly useful, in case one wants to avoid humans wandering in the forest, intense heat or even wildfires, which can potentially put lives and hardware at risk.

The main contributions of this work are:

- A publicly available dataset formed by manually annotated visible and thermal images of two different tree species, taken in different forestry areas;
- The detection of forest trunks in visible and thermal images;
- The study and benchmarking of DL-based object detection models for the detection of forest trunks, in different hardware platforms.

The remainder of this paper is structured as follows. Section 2 presents and describes the state of the art in object detection, mainly the models that were used in this work. Section 3 shows the followed methodology to acquire the data and their processing; the DL models that were used in this work, their training configurations and the evaluation format to assess them are also shown in this section. In Section 4, the results of this work and a discussion are presented. This paper ends with Section 5, where the main discoveries made with this work are described and some future work is proposed.

## 2. Deep Learning-Based Object Detection

This section presents the state-of-the-art of object detection through deep learning methods, highlighting the architectures and models that were used in this work: SSD [46], SSDLite [47], MobileNetV2 [47], ResNet50 [48], Inception-v2 [49], MobileDet [50], and YOLOv4 Tiny [51]. In addition, a literature review of instance segmentation methods was made, as well as a clarification about the preference of using object detection methods instead of segmentation methods for this work.

### 2.1. Single-Shot Detector

A Single-Shot Detector (SSD) [46] is a fast object detector that is based on a feed-forward CNN that generates a set of candidate bounding boxes along with their scores, which assess the presence of an object of interest inside the boxes. This CNN has a base network capable of high-quality image classification called VGG16 [52] and the authors added an auxiliary structure that enables: detection at multiple scales, the prediction of a set of detections using convolutional filters (that are on top of the SSD network), and the association of default bounding boxes with each feature map cell, allowing one to discretize in an efficient manner the possible shapes of the output boxes [46].

### 2.2. MobileNet and SSDLite

MobileNet [53] by itself is an image classification network, but when grouped, for instance, with an SSD, forms a reliable and fast object detection model. This CNN relies on depth-wise separable convolutions—factorized convolutions—that decrease the required computation and the size of the model. Additionally, all convolutional layers of this neural network are followed by a batch normalization and a Rectified Linear Unit (ReLU) non-linearity, except the last fully connected layer which is directly connected to a softmax classification layer. Although MobileNet is a lightweight and fast CNN, the authors also consider two parameters to reduce the model’s size and speed: width multiplier (α), which simply makes the network thinner uniformly, and a resolution multiplier (ρ), which changes the input image resolution and the representation of every layer accordingly [53].

The second version of MobileNet (MobileNetV2) [47] came in 2018 and this version mostly targeted mobile applications, since its basic structure provides memory efficient inferences. The use of linear bottlenecks and inverted residuals improved the performance of the first version. Along with MobileNetV2, the authors proposed a new object detection framework called SSDLite [47]. This novel network is specially aimed at mobile inference and it differs from the original SSD in the regular convolutions that were replaced by separable convolutions in the prediction layers. In the end, SSDLite resulted in a mobile version of SSD with a low parameter count and low computational cost.

In 2019, a third version of MobileNet (MobileNetV3) [54] appeared. MobileNetV3 came with a tuning for mobile phones by combining a network architecture search with a fine-tuning technique that tunes the layers individually rather than globally. From this version, two subversions emerged: MobileNetV3-Large and MobileNetV3-Small, for higher and lower resource usage applications, respectively.

### 2.3. ResNet

ResNet [48] is a framework whose base is residual learning. Typically, feed-forward neural networks are formed by stacked layers, where the outputs of a layer are directly connected to the inputs of the next layer. Residual networks, on the other hand, add shortcut connections to the usual networks that skip a number of layers. In ResNet, these connections have the role of generating identity maps and their outputs are added to the outputs of the network stacked layers [48]. The major advantages of these connections are that they do not add computational complexity nor extra parameters. The authors of ResNet proven that its deep residual network is easy to optimize and an increase in depth does not cause higher training error, instead ResNet achieves accuracy improvements with greater depth.

### 2.4. Inception

Inception [55] is a CNN that was proposed in 2014 whose design is based on stacked Inception modules formed by 1 × 1, 3 × 3 and 5 × 5 convolutions, and a 3 × 3 max-pooling operation. Between each Inception module occasionally appears a max-pooling layer. The result of each operation is then concatenated, forming the module output.

The second version of Inception (Inception-v2) [49] appeared with a batch-normalization feature that helps speed up the training process. This feature adds only two extra parameters per activation and was taken into consideration to tackle the covariate shift phenomenon, which is known to complicate the training process of most machine learning systems. Additionally, the use of batch normalization helped Inception-v2 outperform, with fewer training steps, the state-of-the-art methods in image classification tasks.

Lastly, Inception-v3 [56]—the third version of Inception—appeared with the aim of decreasing the computational complexity of the Inception network. This was achieved by factorizing bigger convolutions such as 5 × 5 and 7 × 7, which are computational expensive, into two stacked 3 × 3 convolutions, for instance. Another factorization operation that the authors considered is an asymmetric one, i.e., an n×n convolutional would be replaced by one n×1 convolutional on top of a 1×n convolutional. These two types of factorization reduce the computational cost of the network.

### 2.5. You Only Look Once

You Only Look Once (YOLO) [57] is a popular object detection system for real-time applications that emerged in 2016. YOLO starts by resizing the input image, then the image is divided into a S × S grid, and each grid cell is responsible for predicting only a single object. After, for each grid cell and by using a single CNN, YOLO predicts B bounding boxes, where each one has a confidence score, and C class probabilities for each box. The final result is a tensor defined as S × S × (B × 5 + C).

At the time of writing this paper, YOLO already accounts 3 new versions: YOLOv2 [58], YOLOv3 [59] and YOLOv4 [51]. The first two versions showed improvements mostly in terms of detection accuracy and speed. The last version, which is also the last official version of YOLO, is currently the object detector presenting highest frame-rate along with accurate detections [51] in the object detection domain. YOLOv4 has a base composed by CSPDarknet53 [60], a intermediary part made by SPP [61] and Path Aggregation Network (PAN) [62], and an head corresponding to YOLOv3 [59]. In this work we used a smaller version of YOLOv4, called YOLOv4 Tiny, that is more suitable for resource-limited hardware applications such as Tensor Processing Units (TPU).

### 2.6. MobileDet

MobileDet [50] is a recent object detector whose main applicability is for mobile accelerators. The authors wanted to study whether the predominant habit of using depth-wise inverted bottlenecks as the main building block in mobile networks should be taken without considering other approaches. They found out that full convolutions have the potential to improve both accuracy and latency when placed in the most appropriate locations inside the network. Such locations can be found through a neural architecture search. As a result, MobileDets achieved very good detection results, outperforming the majority of cutting-edge mobile methods.

### 2.7. Instance Segmentation Review

Instance segmentation is a domain of deep learning that, like object detection, locates and recognizes objects in images but, instead of using bounding boxes, the detection of the objects is carried out by associating a class label to each pixel of the image, resulting in the objects being masked [63].

One of the first CNNs for instance segmentation was proposed in 2014 and it was called R-CNN [64]. This network consists of the combination of AlexNet [21] with a selective search technique. The training procedure of an R-CNN starts by computing class region proposals using a selective search, followed by fine-tuning a pre-trained AlexNet with the region proposals. After, a set of Support Vector Machine (SVM) classifiers are trained with the extracted features from AlexNet, replacing the soft-max classifier that was learned by fine-tuning. Then, using the features learned by AlexNet, a bounding box regressor training is performed for each object class [64]. This network achieved impressive results; nevertheless, it has some drawbacks related to time. The training of R-CNN takes long time, as it is needed to train each stage of a multi-stage pipeline, and the SVM classifiers and the bounding box regressor must also be trained. In terms of testing, R-CNN is also slow due to the fact that AlexNet has to extract features for each object proposal in every image. From these cons, two new improved versions of R-CNN were developed: Fast R-CNN and Faster R-CNN. Fast R-CNN [65] replaced the multi-staged training pipeline of R-CNN with an end-to-end training procedure by performing simultaneously the learning of soft-max classifier and the class bounding boxes regression. This network remains in the region proposals strategy, but a Region Of Interest (ROI) pooling layer was added to extract features for every region proposal [65]. Such changes produced an impact on Fast R-CNN in terms of efficiency. Comparatively with R-CNN, Fast R-CNN reduced by three times and 10 times the training and testing speeds, respectively. Even so, a newer version appeared in 2017: Faster R-CNN [24]. This network differs from Fast R-CNN by the substitution of a selective search by a CNN for production of region proposals named the Region Proposal Network. This addition resulted in improvements in efficiency and accuracy. A further development was made with the introduction of Mask R-CNN [66]. This CNN did not bring any reduction in the computational cost of Faster R-CNN, but it adds a parallel object mask prediction branch to the object bounding box prediction branch of Faster R-CNN. Another advantage of Mask R-CNN is that it can be easily generalized for other tasks beyond segmentation. MaskLab [67] is another instance segmentation framework that improved Faster R-CNN and produces two additional outputs: semantic segmentation and instance centre direction. The authors proposed a technique that removes the duplicate background encoding and the direction prediction is used for separate several instances of the same object. In 2019, some work was developed to improve Mask R-CNN without losing the generality capacity of the network. In this way, Mask Scoring R-CNN [68] appeared. The difference of this network is the ability of scoring its own masks to assess the quality of them. So, a head module named MaskIoU was added that, along with the typical structure of Mask R-CNN, predicts the level of overlapping between the input mask and the ground-truth mask. This approach computes the alignment error among the mask score and the mask quality, improving the performance of the segmentation task by giving priority to better masks predictions. This network even outperformed Mask R-CNN. PAN [62] is an upgraded version of a Feature Pyramid Network (FPN) [69], where the authors applied bottom-up path augmentation, a process that consists of performing localization in lower level layers, leading to shorter paths of information among the lower layers and the top layers. Moreover, they proposed an approach called adaptive feature pooling that generates a relationship between a grid of features and features at all levels. These techniques introduced a small overhead in terms of computational cost and are easily implemented. Lastly, a fast and simple model that was designed for real-time instance segmentation is YOLACT [70]. This model is constructed in a fully convolutional topology and is capable of running at 33 frames per second. To achieve such a result, the authors divided the image segmentation into two subprocesses: the generation of prototype masks and prediction of mask coefficients for every instance mask; then, the final instance masks are produced by combining, linearly, the prototype masks with the coefficients.

Instance segmentation, despite being a very interesting approach that can totally segment an object in all of its extension, still has some drawbacks: the training and testing can be very slow, some models are very difficult to optimize, the majority of the models are not suited for real-time applications, the need for large storage memory and high computational power. These factors were considered and had weight in the decision not to use methods of this type in this work.

## 3. Materials and Methods

In this section, the study areas in which the data were collected are presented, as well as the species of forest trees existing in the dataset, and the cameras that were used to make the local footage. Additionally, the dataset augmentation operations that were made before any training are explained and the model configuration for training and evaluation metrics are addressed.

### 3.1. Study Areas and Forests, and Image Acquisition Methodology

The collection of images was performed in Portugal in three different areas: Valongo (41°11${}^{\prime }$22.09${}^{\prime \prime }$ N, 8°29${}^{\prime }$55.54${}^{\prime \prime }$ W), Vila do Conde (41°21${}^{\prime }$14.22${}^{\prime \prime }$ N, 8°44${}^{\prime }$30.66${}^{\prime \prime }$ W) and Lobão (40°59${}^{\prime }$05.10${}^{\prime \prime }$ N, 8°29${}^{\prime }$17.41${}^{\prime \prime }$ W). These three forestry areas are mainly composed of two tree species: eucalyptus and pinus.

**Figure 1:**
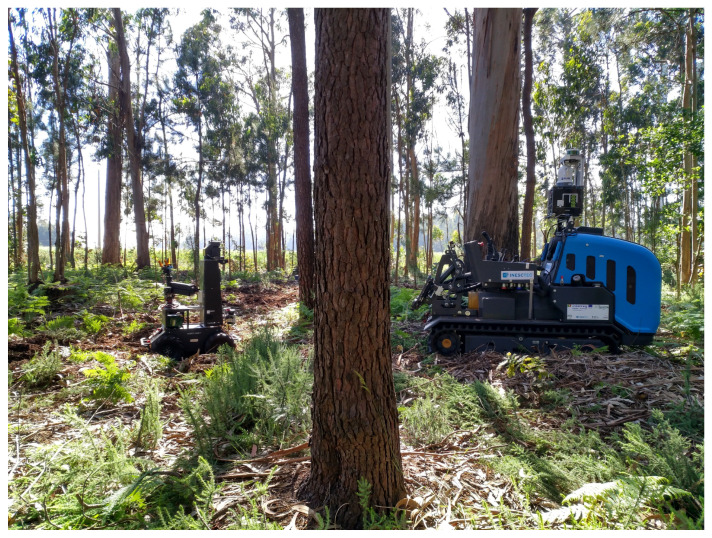
Robotic platforms used to acquire the forest images: on the left side is shown AgRobV16 and on the right side is shown AgRobV18.

The image acquisition was realized with four cameras: GoPro Hero6 (https://gopro.com/en/gb/update/hero6, accessed at 10 July 2021), FLIR M232 (https://www.flir.eu/products/m232, accessed at 10 July 2021), ZED Stereo (https://www.stereolabs.com/zed, accessed at 10 July 2021) and Allied Mako G-125 (https://www.alliedvision.com/en/products/cameras/detail/Mako%20G/G-125.html, accessed at 10 July 2021). The images from Valongo were gathered with ZED Stereo camera (used as monocular camera) mounted on AgRobV16—a ground robot that is presented in Figure 1, on left side; the images from Vila do Conde were acquired with the Allied Mako G-125 also mounted on AgRobV16; FLIR M232 and GoPro Hero6 took pictures in the forest of Lobão: the former was mounted on AgRobV18—another ground robot that is presented in Figure 1, on the right side—and the latter was transported by hand. The resolution of the cameras and the corresponding spectrum of their images are presented further in Table 1.

### 3.2. Dataset Preparation, Augmentation and Split

**Figure A1:**
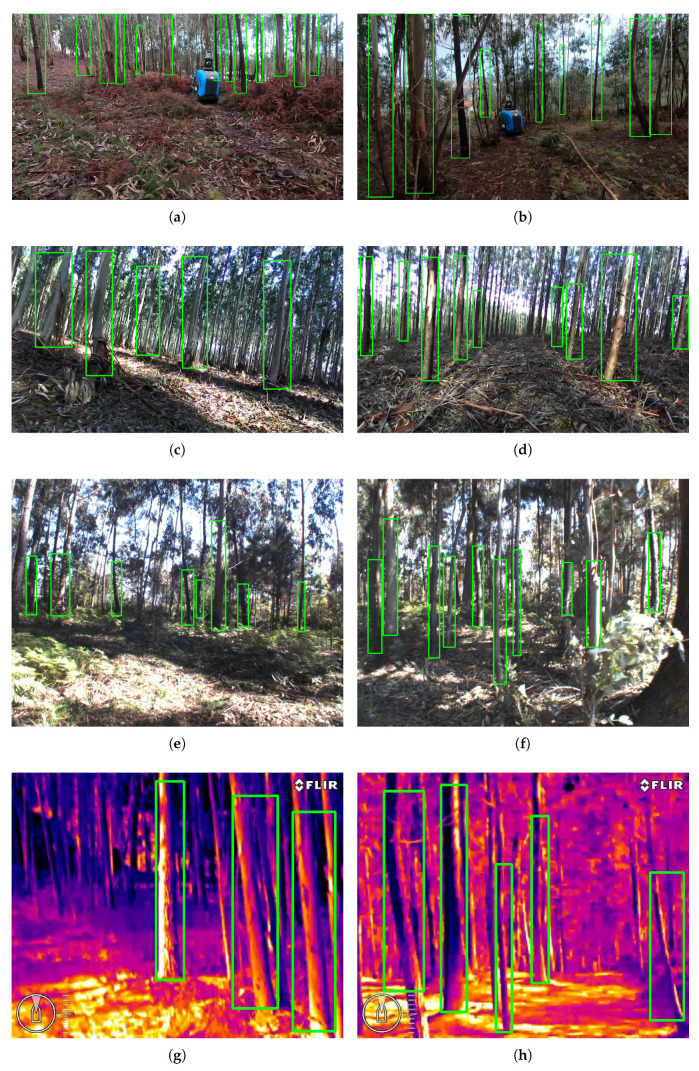
Examples of images with manual annotations: (**a**,**b**) are visible images from Lobão, (**c**,**d**) are visible images from Valongo, (**e**,**f**) are visible images from Vila do Conde, and (**g**,**h**) are thermal images from Lobão.

The images acquired in-site were pre-selected according to their suitability for the task at hand—detecting forest trunks—i.e., images presenting any kind of defects such as excessive blur or sun-related incandescent effects were eliminated. Then, they were manually annotated using Computer Vision Annotation Tool (CVAT) (https://github.com/openvinotoolkit/cvat, accessed at 10 July 2021) with the Pascal Visual Object Classes (VOC) format, a commonly known format related to Pascal VOC Challenge [71] where each image file has a linked Extensible Markup Language (XML) file that holds the annotations (examples of annotated images belonging to the dataset are shown in Appendix A in Figure A1).

**Figure 2:**
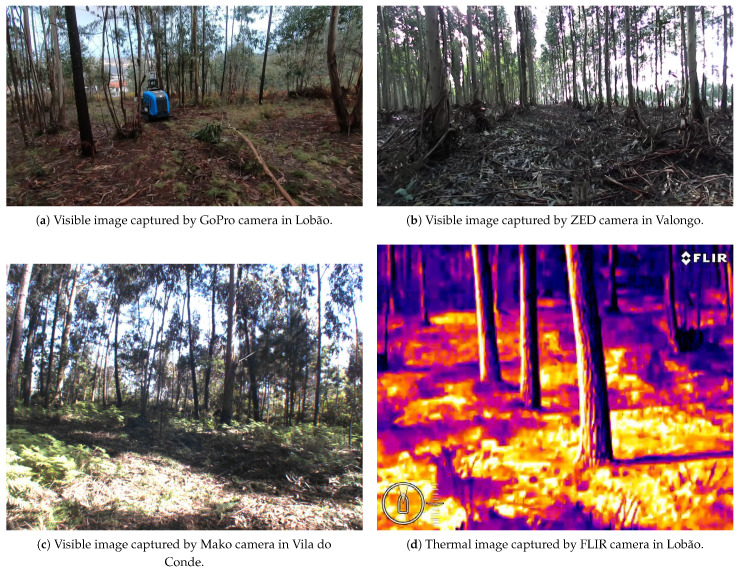
Illustrative images captured by the four cameras in different locations: (**a**) GoPro, (**b**) ZED, (**c**) Mako, and (**d**) FLIR.

The original dataset resulting from the pre-selection is formed by 2895 images. The characteristics of the images are presented in Table 1. An important aspect to be highlighted is that the dataset not only contain images of visible spectrum, but also thermal images. Figure 2 shows 4 distinct images taken by the cameras in different forests.

Since the DL models require large amounts of data to achieve higher levels of accuracy, the original dataset was augmented. The augmentation operations that were performed on the original dataset are presented and described in Table 2.

**Figure 3:**
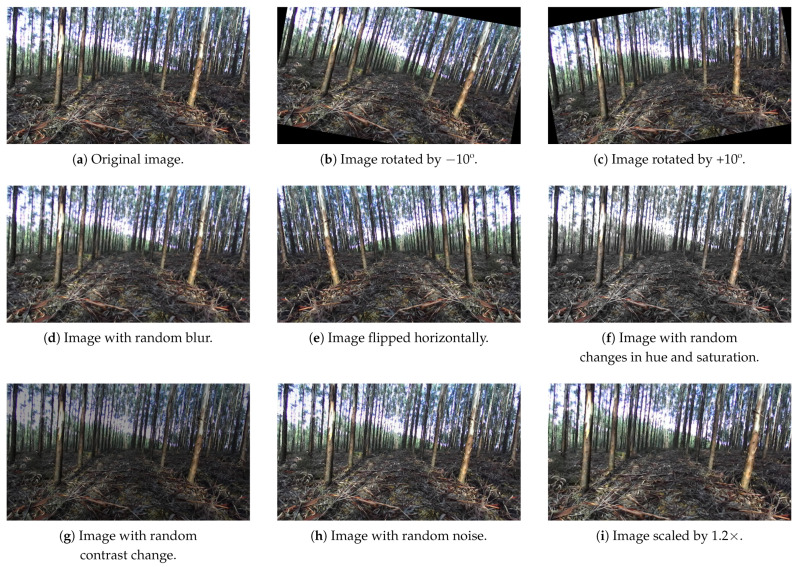
Augmentation operations in one image: (**a**) original image, (**b**) −10° rotation, (**c**) +10° rotation, (**d**) random blur, (**e**) horizontal flip, (**f**) random hue and saturation changes, (**g**) random contrast changes, (**h**) random noise addition, and (**i**) 1.2× scaling.

Figure 3 presents the application of these augmentation operations to an image of the dataset. In total, they formed a set of seven operations and eight transformations. Therefore, it was expected that the number of images in the augmented dataset would be 8×2895+2895=26,055; however, we also removed the non-annotated images (images that did not contain any good trunk to annotate) figuring in the original dataset, because these could compromise the model’s learning performance. Summing up, there were 205 images in the original dataset that were unconsidered, so the augmented dataset was in turn formed by 9×(2895−205)=24,210 images. The original and augmented datasets were made publicly available (https://doi.org/10.5281/zenodo.5213825, accessed at 17 August 2021).

Before start training the DL models, the augmented dataset was split into three subsets: train, validation and test. The train subset is used for training, the validation subset is utilized to assess the training process and to verify if the model does not excessively over-fit, and the test subset is used to test and evaluate the models after the training. The benchmarking of the DL models used in this study will be made with the test subset. The split ratios that were chosen were 80%, 5% and 15% for the train, validation and test subsets, respectively. Hence, this division originated a train subset of 19,369 images, a validation subset of 1210 images and a test subset of 3631 images. The percentage of the validation subset was this small because this made the training process highly efficient; larger ones made the training time increase considerably.

### 3.3. DL Models Configuration and Training

The DL models selected in this work were: SSD MobileNetV2, SSD Inception-v2, SSD ResNet50, SSDLite MobileDet and YOLOv4 Tiny. All the models were trained using transfer learning with pre-trained weights from the Microsoft Common Objects in Context (COCO) dataset [72].

The SSD-based DL models were collected from the model zoo of TensorFlow version 1 (https://github.com/tensorflow/models/blob/master/research/object_detection/g3doc/tf1_detection_zoo.md, accessed at 10 July 2021), and they were trained using TensorFlow Object Detection API (https://github.com/tensorflow/models/tree/master/research/object_detection, accessed at 10 July 2021) with Google Colaboratory (https://colab.research.google.com/, accessed at 10 July 2021) due to the easy access to Graphics Processing Unit (GPU), which makes the training process faster. To train YOLOv4 Tiny, was used Darknet (https://github.com/AlexeyAB/darknet, accessed at 10 July 2021), also with Google Colaboratory.

The details related to the training of the DL models are shown in Table 3. The batch sizes are different because the models have different architectures, and sometimes a bigger batch size would make Google Colaboratory to crash. With respect to the learning rates, were used the pre-defined ones.

The training processes were stopped when the training and validation loss curves converged, i.e., when the curves presented minimal variation (≤5%). The SSD MobileNetV2 converged after 100,000 steps of training, SSD Inception-v2 converged after 80,000 steps, SSD ResNet50 converged after 120,000 steps, SSDLite MobileDet converged after 90,000 steps and YOLOv4 Tiny converged after 25,000 steps, as can be seen in Table 3.

### 3.4. DL Models Evaluation Experiments, Conditions and Metrics

After training, the models were evaluated on the images of the test subset. Using this subset of images, we have made three different experiments:

1. Division of the test data-subset into four sub-subsets (visible images in Lobão, visible images in Valongo, visible images in Vila do Conde and thermal images) and run inference in each sub-subset;
2. Division of the test subset into two sub-subsets (visible images and thermal images) and run inference in each sub-subset;
3. Run inference in the entire test subset—with visible and thermal images mixed, and with mixed forestry places.

The first experiment (experiment #1) will allow us to study the models performances in single forests and to evaluate if there are any specific forest that reduces the models’ detection competence. The second experiment (experiment #2) will help us understand the impact that thermal images can cause to the models performance, and whether or not is possible to detect forest trunks in thermal images with high level of precision. The third and last experiment (experiment #3) aims at a complete benchmark of the models using all kinds of images, taken in different forestry areas. The evaluation sets that are part of these experiments are detailed in Table 4. The names of the evaluation sets that are shown in the table will be used later on to present and discuss the results collected from the image sets.

It is important to consider that all methods have a Non-Maximum Suppression (NMS) post-processing operation configured with a confidence threshold of 30% and an overlapping threshold of 60%. This way, only detections with confidence scores above 30% are considered for the NMS step. Additionally, all models were evaluated in terms of accuracy, and inference time in two different hardware platforms, one of them is based on a four-core Central Processing Unit (CPU) and the other one has a GPU with a compute capability of 7.5. The two platforms are specified in Table 5.

The metrics that are usually used to evaluate object detection methods are based on True Positives (TP), correctly detected objects, False Positives (FP), incorrectly detected objects, and False Negatives (FN), which represents incorrectly undetected objects. From these concepts, two metrics called Precision (P) and Recall (R) emerge. Precision measures how many detections are objects, and depends on the number of True Positives and False Positives, as can be seen in (1). Recall measures how many objects are detected, and depends on the True Positives and False Negatives, as can be seen in (2).

(1) $$P=\frac{\mathrm{TP}}{\mathrm{TP}+\mathrm{FP}}$$

(2) $$R=\frac{\mathrm{TP}}{\mathrm{TP}+\mathrm{FN}}$$

Based on the two previous metrics, Precision × Recall curve is another way of measuring the performance of a detector and corresponds to a trade-off between Precision and Recall [73]. This curve is useful for computing Area Under the Curve (AUC). This metric tells us that our method is good if it presents an high AUC. To maximize AUC, the Precision must stay high while the Recall increases. However, measuring AUC is quite difficult due to the zigzag behaviour of Precision × Recall curve, so a metric called Average Precision (AP) is capable of calculating it, summarizing the curve shape by the average of the maximum Precision values at each Recall level [73], interpolating through all points in the following fashion:

(3) $$\mathrm{AP}=\sum_{n}\left(R_{n+1}-R_{n}\right)P_{interp}\left(R_{n+1}\right),$$

with

(4) $$P_{interp}\left(R_{n+1}\right)=\max_{\tilde{R}:\tilde{R}\geq R_{n+1}}P\left(\tilde{R}\right)$$

where $P(\tilde{R})$ is the Precision measured at Recall $\tilde{R}$.

Another metric to assess and compare the models performances is F1 score. F1 score is simply the harmonic mean of Precision and Recall, which allows maximizing these two at the same time, and is defined by:

(5) $$F1=2\times \frac{P\times R}{P+R}$$

In object detection tasks, the previous metrics require another metric to distinguish a correct detection from an incorrect one. That metric is Intersection over Union (IoU). This metric is defined in (6) and it measures the overlapping area between a ground-truth bounding box ($B_{gt}$) and a detected bounding box ($B_{det}$). Then, this area is compared with a given threshold and if it lies above or under the threshold, the detection is considered as correct or incorrect, respectively [73,74].

(6) $$\mathrm{IoU}=\frac{area(B_{gt}\cap B_{det})}{area(B_{gt}\cup B_{det})}$$

## 4. Results and Discussion

This section presents the results, and their discussion, gathered in this work, which are related to the performance aspects of the trained models for each experiment presented by Table 4 in Section 3.4. All models were evaluated according to AP, F1 score and the Precision × Recall curve. Additionally, they were also tested on CPU and GPU to assess their inference times. To evaluate the detection performance of the models, an IoU threshold of 50% was considered, as this is one of the most common values [73]. A detail that should be mentioned is that all models were trained and tested using their default input resolutions. Lastly, and for simplicity, we consider the confidence threshold that includes all detections as 0%, but in our case this threshold is actually 30%, since we ignored the predictions with confidence scores below this last value, as was mentioned in Section 3.4.

### 4.1. Results of Experiment #1

Experiment #1 is about performing inference with the trained models in each of four evaluation sets: visible images from Vila do Conde (VC_VISIBLE), thermal images from Lobão (LB_THERMAL), visible images from Lobão (LB_VISIBLE) and visible images from Valongo (VG_VISIBLE). The evaluation results are focused on the AP and F1 score that the models achieved on the evaluation sets previously mentioned. The results of this experiment are presented in Table 6.

From Table 6, it can be seen that in general all models achieved the best AP and F1 results in the VG_VISIBLE evaluation set. In the LB_THERMAL and VC_VISIBLE evaluation sets, the models attained similar results in each set, and the worst results were collected when the models ran in the LB_VISIBLE set. In addition to that, YOLOv4 Tiny was the best detector in every evaluation set, and SSDLite MobileDet was the worst. Lastly, it should be concluded that the data subset that provokes more errors in the models is the one with visible images from Lobão.

### 4.2. Results of Experiment #2

The objective of experiment #2 is to run inference using the trained models on the two evaluation sets of the second experiment: thermal images only (ONLY_THERMAL) and visible images only (ONLY_VISIBLE). The evaluation results are focused on the AP and F1 score that the models attained on the aforementioned evaluation sets. The results of this experiment are presented in Table 7.

From Table 7, it can be seen that the results obtained from experiment #2 are equally distributed by the evaluation sets: some models collected clearly better AP and F1 results on the thermal image-based set (ONLY_THERMAL), for instance, SSD MobileNetV2 and SSDLite MobileDet; others got clearly better AP and F1 results on the visible image-based set (ONLY_VISIBLE), such as YOLOv4 Tiny; lastly, SSD Inception-v2 and SSD ResNet50 presented very similar results for both evaluation sets. Again, YOLOv4 Tiny was the best detector in the two evaluation sets, and SSDLite MobileDet was the worst. The similarity of the results gathered on the two evaluation sets proves that the detection of forest tree trunks in thermal images is surely possible and was performed with high values of AP and F1 in this work.

### 4.3. Results of Experiment #3

Experiment #3 consisted in using the ALL evaluation set (detailed in Table 4) to run inference with the trained models. This evaluation set is equal to the test subset mentioned in Section 3.2 and includes visible and thermal images. The results obtained from this experiment will be analyzed focusing on the detection performance of the models, on the impact of increasing the confidence level in the AP and F1 of the models, and on the temporal performance of the models in different hardware platforms (CPU and GPU).

#### 4.3.1. Detection Performance of the Models

Table 8 presents the AP and F1 results, where all predictions were considered, i.e., the values present in the table are the ones related to a confidence threshold of 0%.

From Table 8, it can be said that notoriously YOLOv4 Tiny is by far the best trunk detector in AP and F1 on GPU, as it gathered 89.84% AP and 89.37% F1. The second best trunk detector is SSD ResNet50, which presented a 78.19% AP and 84.75% F1. Next is SSD Inception-v2 with a 75.29% AP and 83.98% F1, followed by SSD MobileNetV2 that achieved 72.68% AP and 80.74% F1. Lastly, SSDLite MobileDet was the detector that presented the worst AP (68.08%) and F1 (73.53%) performances.

**Figure 4:**
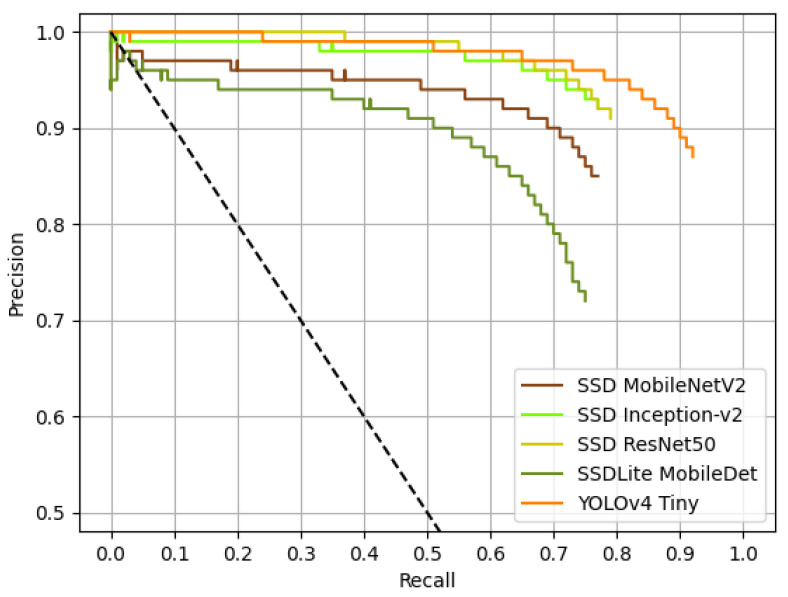
Precision × Recall curves of all models for a 0% confidence level. The dashed black curve represents the linear interpolation between 1.0 Precision and 1.0 Recall.

The AP values of the previous table can be correlated with the AUC of the detectors. For this, in Figure 4, the Precision × Recall curves of the detectors are presented.

**Figure 5:**
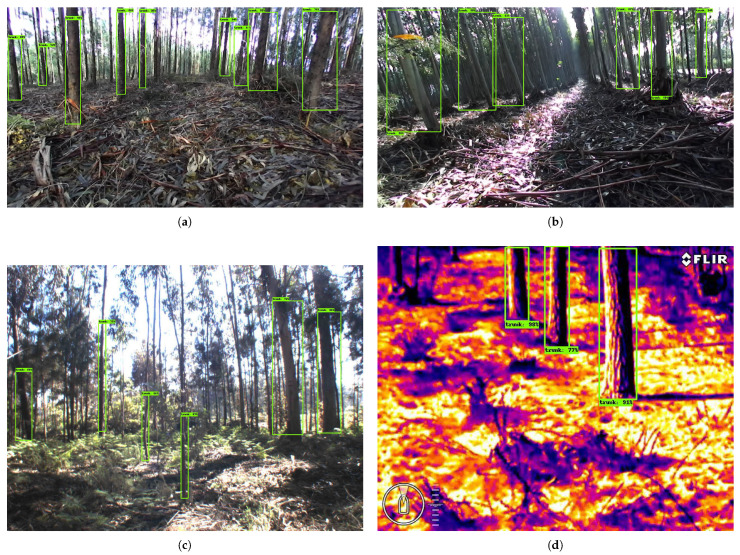
Examples of detection using SSD ResNet50 in (**a**–**c**) three visible images and (**d**) in one thermal image.

The previous figure tells us about the accordance between the previously found AP values with the AUC of the detectors, since YOLOv4 Tiny have the best AP and, according to Figure 4, also seem to have the highest AUC. The same can be said to remaining detectors and the correlation of their APs and AUCs: SSD ResNet50 seem to have the second highest AUC, followed by SSD Inception-v2, SSD MobileNetV2 and SSDLite MobileDet. Figure 5 shows four detection examples using SSD ResNet50.

#### 4.3.2. AP and F1 versus Confidence

**Figure 6:**
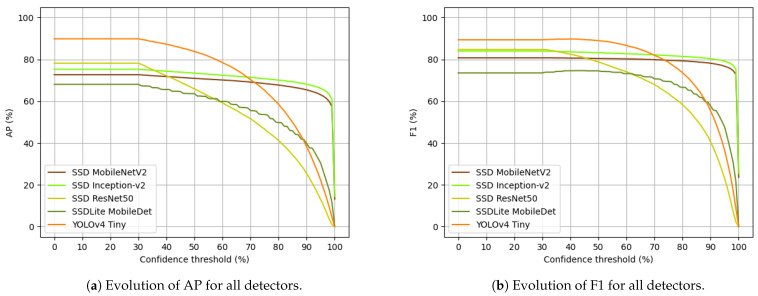
Results of the evolution of AP (**a**) and F1 (**b**) metrics over the full range of confidence levels.

To verify the evolution of both AP and F1 score over the full interval of confidence (from 0% to 100%), we present Figure 6.

From Figure 6, YOLOv4 Tiny is the best forest trunk detector, in AP and F1, with nearly 68% confidence. From that point onwards, SSD Inception-v2 was the best detector, followed by SSD MobileNetV2. SSD ResNet50 was the second best detector until around 35% confidence, from which it started decreasing (in AP and F1) and, when it reached the 60% confidence level, it became the worst detector overall, falling behind SSDLite MobileDet, that in turn until this point was the worst trunk detector.

In general, all detectors showed decreasing AP and F1 when the confidence level increased. To assess this deterioration of the results, we checked the absolute decrease the models suffered from 0% to 95% confidence. The absolute values are presented in Table 9.

The detectors that showed smallest AP and F1 decreases from 0% to 95% were SSD Inception-v2 with 9.13% in AP and 4.86% in F1, and SSD MobileNetV2 with 9.62% in AP and 4.06% in F1—proving that they are reliable forest trunk detectors, since the confidence increment does not affect them massively as it does to the remaining detectors. SSDLite MobileDet is the third model in terms of its AP and F1 reduction—it decreased 37.68% and 25.89% in AP and F1, respectively. Lastly, YOLOv4 Tiny and SSD ResNet50 were the detectors that presented major decrements: YOLOv4 Tiny decreased 67.61% in AP and 52.94% in F1, and SSD ResNet50 decreased 65.19% in AP and 61.72% in F1.

#### 4.3.3. Temporal Results of Inference

To assess the speed of the detectors, the inference times of the experiments made on the two types of hardware (CPU and GPU) are shown in Table 10, where the time values correspond to average inference times per image.

From the previous table, it can be seen that, on CPU, the fastest model was SSD MobileNetV2 with an inference time of 58 ms. The slowest method on CPU was SSD ResNet50, taking on average 1789 ms to compute a detection result; on GPU, the detector that takes the least time to run through each image was YOLOv4 Tiny with 9 ms. Again, SSD ResNet50 was time-wise the worst method, averaging 50 ms per image on GPU.

### 4.4. Discussion

The results gathered from the experiments made in this work show that the detection of forest tree trunks at ground level can be performed with high accuracy and reliability. Additionally, it was proven that with right amount of relevant data, several DL models can be used for this task, as they are capable of yielding impressive detection results, even in thermal images, as was proven with experiment #2. The remainder of this section presents the discussion of the results obtained in experiment #3.

The work made in [22] can be compared with ours, as the authors assessed the detection of trees at ground level, although their images were taken from the street, instead of being captured in forestry locations. Nonetheless, comparing our results to [22], it can be said that we obtained extremely good results considering the best model that was used (YOLOv4 Tiny with 89.84% AP and 89.37% F1) and considering our testing conditions: the models were tested on a test set made of by 3632 images in forestry areas, that by itself makes the detection even more difficult due to strong shadowing (an example of this is shown in Figure 5c) and the existence of many more trees, whereas in [22] the test set only contained 89 images, certainly in each image there are fewer trees than in one of our test images, and the images are from the street, which makes the trees easier to detect. Even so, the authors in [22] claimed that they achieved an AP of 98% using YOLOv2 with a pre-trained ResNet50 as the feature extractor. In [25], the authors used a occlusion-aware R-CNN for detecting trees in street images. They stated that their implementation achieved the best miss rate of 20.62%. So, even though the evaluation metric is not the same as ours, we believe that comparing to this work, our models behaved excellent given that our ALL test dataset is around seven times larger than theirs.

An important thing to mention is that, regarding the performance drop that YOLOv4 Tiny, SSDLite MobileDet and SSD ResNet50 suffered with the increase in confidence, it can be fought by training these detectors longer, as more training time can increase a detector confidence in its detections.

To compare the models in global terms, three different variables were used: accuracy, speed and memory. The accuracy was assessed using the results from Table 8; to evaluate the speed, Table 10 was used; and memory was calculated considering the occupied memory by the models. For the evaluation, a grading system from zero to four (0–4) was considered. So, if a model was the most accurate, the fastest and occupied the least amount of memory, it would obtain the grade four in each variable. On the contrary, if a model was the least accurate, the slowest and occupied the most memory, it would be graded zero in each variable. Table 11 presents a comparison of the models according to the aforementioned grade system.

After analyzing the previous table, one can say that, overall, YOLOv4 Tiny was the best model and SSD ResNet50 the worst one. In terms of accuracy, YOLOv4 Tiny was the best and SSDLite MobileDet the worst; in terms of speed, YOLOv4 TIny was the best and SSD ResNet50 the worst; and in terms of memory, SSDLite MobileDet was the best and SSD ResNet50 the worst.

## 5. Conclusions

In this work, a benchmarking study was made aiming at the image-based detection of forest tree trunks at ground level using deep learning methods, specifically object detection CNNs. The tree trunk detection was carried out not only on visible images, but also on thermal images, an approach that at first was not guaranteed to work. The use of thermal images allows the execution of in-field forestry operations during the day and night, meaning that this is a very important and innovative advance in the forestry domain, for navigation and inventory purposes. For this, a dataset composed of visible and thermal images was built, totalling 2895 images that were taken by four different cameras in three different places, and comprising two tree species: eucalyptus and pinus. All images were manually annotated following the Pascal VOC format [71], and the original dataset was also augmented, as DL models require large amounts of data to achieve better performances, resulting in an augmented dataset of 24,210 images.

The DL detectors that were used in this work were: SSD MobileNetV2, SSD Inception-v2, SSD ResNet50, SSDLite MobileDet and YOLOv4 Tiny, and all of them were trained using transfer learning from pre-trained weights from the COCO dataset [72]. After training, three experiments were conducted using the trained models. The first experiment allowed us to conclude that the visible images from Lobão forest induce the most errors to the models; with the second experiment, it was possible to conclude that the detection of forest tree trunks in thermal images is possible and can be achieved with a high level of precision. The third and last experiment corresponds to a global evaluation of the models using different types of images from different forests. More specifically, this experiment aimed at evaluating the detection performance of the models with some metrics and their temporal performances by running inference on different hardware platforms: CPU and GPU. The results of the third experiment showed that YOLOv4 Tiny was the model that attained the highest AP and F1, with 89.84% and 89.37%, respectively. On the other hand, SSDLite MobileDet was the method that yielded the lowest results with a AP of 68.08% and an F1 of 73.53%. With respect to the variation of AP and F1 of the methods with the increase in confidence, SSD Inception-v2 and SSD MobileNetV2 were the best detectors, presenting the lowest variations among all detectors: from 0% to 95% confidence, SSD Inception-v2 decreased 9.13% in AP and 4.86% in F1, and SSD MobileNetV2 decreased 9.62% in AP and 4.06% in F1. The method that was more affected by the increasing confidence levels was SSD ResNet50, with a decrease of 65.19% and 61.72% in AP and F1, respectively. In terms of inference time per image, SSD MobileNetV2 was the fastest model running on CPU with an average inference time of 58 ms; SSD ResNet50 was the slowest one, taking 1789 ms to complete an inference. On GPU, YOLOv4 Tiny took 8 ms on average to infer, being the fastest in this hardware platform, whilst SSD ResNet50 was again the model taking more time to run inference with an average time of 50 ms.

After this work, it may be concluded that YOLOv4 Tiny is the best model, from the set of models used in this work, for detecting forest trunks if confidence levels could be ignored; otherwise, SSD Inception-v2 or SSD MobileNetV2 are the detectors to use.

Future work includes, studying the impact of using the same input resolution in the models; training the models with quantization-aware procedures to further enable running them on edge devices and TPUs, as the inference time on these can be even lower, and it would be interesting to compare those results to the ones presented in this work; increasing the dataset by the addition of depth images, since this type of image is robust to light variations which happens a lot in forests and can compromised the performance of the detectors; increasing the dataset with image samples containing more forest objects to be detected instead of just tree trunks; integrating these models in existing forestry robots to perform autonomous navigation or inventory-related tasks relying on the detections of the models; perform experiments using images acquired at night, with artificial illumination, to study fluorescent techniques to detect tree trunks; and train and test more models to conduct an even broader study and benchmark.

## Figures and Tables

**Table 1:** Original dataset characteristics.

Camera	Image Spectrum	Resolution	Footage Local	Number of Images
GoPro Hero6	Visible	1920 × 1080	Lobão	715
FLIR M232	Thermal	640 × 512	Lobão	866
ZED Stereo	Visible	1280 × 720	Valongo	847
Allied Mako G-125	Visible	1292 × 964	Vila do Conde	467
**Total**				2895

**Table 2:** Augmentation operations.

Operation	Value	Description
Blur	Random	Blur the image
Flip	-	Flip the image horizontally
HueSatur	Random	Change image’s hue and saturation levels
Multiply	Random	Change image’s contrast level
Noise	Random	Add Gaussian noise to the image
Rotation	−10°	Rotate the image −10°
Rotation	+10°	Rotate the image +10°
Scale	1.2×	Scaling the image

**Table 3:** Training details of the DL models.

Model	Learning Rate	Batch Size	Training Steps
SSD MobileNetV2	0.004	24	100,000
SSD Inception-v2	0.004	24	80,000
SSD ResNet50	0.004	8	120,000
SSDLite MobileDet	0.004	32	90,000
YOLOv4 Tiny	0.0026	64	25,000

**Table 4:** Evaluation image sub-subsets for DL inference for each evaluation experiment.

Experiment	Evaluation Set Name	Forests Involved	Image Spectrum	Number of Images
#1	VC_VISIBLE	Vila do Conde	Visible	646
	LB_THERMAL	Lobão	Thermal	915
	LB_VISIBLE	Lobão	Visible	939
	VG_VISIBLE	Valongo	Visible	1131
#2	ONLY_THERMAL	Lobão	Thermal	915
	ONLY_VISIBLE	Lobão, Valongo, Vila do Conde	Visible	2716
#3	ALL	Lobão, Valongo, Vila do Conde	Visible, Thermal	3631

**Table 5:** Hardware platforms specifications for DL inference.

Processing Unit	Platform	Memory
CPU—Intel i7 4 × 2.40 GHz	HP Notebook personal computer	4 GB
GPU—NVIDIA Tesla T4	Google Colaboratory hosted runtime	12 GB

**Table 6:** Evaluation results for experiment #1 in terms of AP and F1 score considering all detections (confidence threshold of 0%).

Evaluation Set	Model	AP (%)	F1 (%)
VC_VISIBLE	SSD MobileNetV2	73.19	80.56
	SSD Inception-v2	76.40	84.72
	SSD ResNet50	75.90	82.69
	SSDLite MobileDet	71.92	74.76
	YOLOv4 Tiny	90.96	90.08
LB_THERMAL	SSD MobileNetV2	77.07	83.02
	SSD Inception-v2	74.93	83.96
	SSD ResNet50	78.63	81.66
	SSDLite MobileDet	73.45	74.71
	YOLOv4 Tiny	86.73	85.82
LB_VISIBLE	SSD MobileNetV2	64.36	71.40
	SSD Inception-v2	66.90	77.93
	SSD ResNet50	70.61	79.90
	SSDLite MobileDet	57.93	65.98
	YOLOv4 Tiny	84.76	85.08
VG_VISIBLE	SSD MobileNetV2	77.10	84.34
	SSD Inception-v2	80.88	87.71
	SSD ResNet50	83.87	89.21
	SSDLite MobileDet	72.57	78.01
	YOLOv4 Tiny	93.59	93.23

**Table 7:** Evaluation results for experiment #2 in terms of AP and F1 score considering all detections (confidence threshold of 0%).

Evaluation Set	Model	AP (%)	F1 (%)
ONLY_THERMAL	SSD MobileNetV2	77.07	83.02
	SSD Inception-v2	74.93	83.96
	SSD ResNet50	78.63	81.66
	SSDLite MobileDet	73.45	74.71
	YOLOv4 Tiny	86.73	85.82
ONLY_VISIBLE	SSD MobileNetV2	72.04	80.40
	SSD Inception-v2	75.34	83.99
	SSD ResNet50	78.20	85.25
	SSDLite MobileDet	67.29	73.34
	YOLOv4 Tiny	90.27	89.93

**Table 8:** Results of AP and F1 obtained from experiment #3 considering all detections (confidence threshold of 0%).

Evaluation Set	Model	AP (%)	F1 (%)
ALL	SSD MobileNetV2	72.68	80.74
	SSD Inception-v2	75.29	83.98
	SSD ResNet50	78.19	84.75
	SSDLite MobileDet	68.08	73.53
	YOLOv4 Tiny	89.84	89.37

**Table 9:** AP and F1 absolute reduction from 0% to 95% confidence.

Model	AP (%)	F1 (%)
SSD MobileNetV2	9.62	4.06
SSD Inception-v2	9.13	4.86
SSD ResNet50	65.19	61.72
SSDLite MobileDet	37.68	25.89
YOLOv4 Tiny	67.61	52.94

**Table 10:** Average inference times of the detectors on different hardware platforms.

Model	CPU (ms)	GPU (ms)
SSD MobileNetV2	58	15
SSD Inception-v2	118	19
SSD ResNet50	1789	50
SSDLite MobileDet	85	17
YOLOv4 Tiny	95	9

**Table 11:** Global comparison between the models.

Model	Accuracy	Speed	Memory	Overall
SSD MobileNetV2	1	3	3	7
SSD Inception-v2	2	1	1	4
SSD ResNet50	3	0	0	3
SSDLite MobileDet	0	2	4	6
YOLOv4 Tiny	4	4	2	10

## Data Availability

The datasets presented in this study are publicly available in Zenodo at https://doi.org/10.5281/zenodo.5213825, accessed at 17 August 2021.

## Abbreviations

The following abbreviations are used in this manuscript:

ANN	Artificial Neural Network
AP	Average Precision
AUC	Area Under the Curve
CCD	Charge-Coupled Device
CNN	Convolutional Neural Network
COCO	Common Objects in Context
CPU	Central Processing Unit
CVAT	Computer Vision Annotation Tool
DL	Deep Learning
FN	False Negatives
FP	False Positives
FPN	Feature Pyramid Network
GPS	Global Positioning System
GPU	Graphics Processing Unit
IoU	Intersection over Union
KNN	K-Nearest Neighbours
NMS	Non-Maximum Suppression
PAN	Path Aggregation Network
R-CNN	Regions with CNN features
ReLU	Rectified Linear Unit
R-FCN	Region-based Fully Convolutional Networks
ROI	Region Of Interest
RTK	Real-Time Kinematic
SLAM	Simultaneous Localization And Mapping
SSD	Single-Shot MultiBox Detector
SVM	Support Vector Machine
TP	True Positives
TPU	Tensor Processing Unit
UAV	Unmanned Aerial Vehicle
UGV	Unmanned Ground Vehicle
VOC	Visual Object Classes
XML	Extensible Markup Language
YOLO	You Only Look Once
